# Undetectable Production of the VIM-1 Carbapenemase in an *Atlantibacter hermannii* Clinical Isolate

**DOI:** 10.3389/fmicb.2021.741972

**Published:** 2021-12-20

**Authors:** Delphine Girlich, Rémy A. Bonnin, Alexis Proust, Thierry Naas, Laurent Dortet

**Affiliations:** ^1^LabEx Lermit, Faculty of Medicine, INSERM UMR 1184—Team RESIST, Université Paris-Sud, Université Paris-Saclay, Le Kremlin-Bicêtre, France; ^2^Associated French National Reference Center for Antibiotic Resistance: Carbapenemase-Producing Enterobacteriaceae, Le Kremlin-Bicêtre, France; ^3^Department of Hormonal Biochemistry, Hôpital de Bicêtre, Assistance Publique—Hôpitaux de Paris, Le Kremlin-Bicêtre, France; ^4^Bacteriology-Hygiene Unit, Assistance Publique - Hôpitaux de Paris, Bicêtre Hospital, Le Kremlin-Bicêtre, France

**Keywords:** β-lactamase, carbapenemase, transcription, *Enterobacter*, plasmid

## Abstract

The differential expression of VIM-1 in *Atlantibacter hermannii* WEB-2 and *Enterobacter hormaechei* ssp. *hoffmannii* WEB-1 clinical isolates from a rectal swab of a hospitalized patient in France was investigated. *A. hermannii* WEB-2 was resistant to all β-lactams except carbapenems. It produced ESBL SHV-12, but the Carba NP test failed to detect any carbapenemase activity despite the production of VIM-1. Conversely, *E. hormaechei* WEB-1, previously recovered from the same patient, was positive for the detection of carbapenemase activity. The *bla*_VIM–1_ gene was located on a plasmid and embedded within class 1 integron. Both plasmids were of the same IncA incompatibility group and conferred the same resistance pattern when electroporated in *Escherichia coli* TOP10 or *Enterobacter cloacae* CIP7933. Quantitative RT-PCR experiments indicated a weaker replication of pWEB-2 in *A. hermannii* as compared to *E. hormaechei*. An isogenic mutant of *A. hermannii* WEB-2 selected after sequential passages with increased concentrations of imipenem possessed higher MICs for carbapenems and cephalosporins including cefiderocol, higher levels of the *bla*_VIM–1_ gene transcripts, and detectable carbapenemase activity using the Carba NP test. Assessment of read coverage demonstrated that a duplication of the region surrounding *bla*_VIM–1_ gene occurred in the *A. hermannii* mutant with detectable carbapenemase activity. The lack of detection of the VIM-1 carbapenemase activity in *A. hermannii* WEB-2 isolate was likely due to a weak replication of the IncA plasmid harboring the *bla*_VIM–1_ gene. Imipenem as selective pressure led to a duplication of this gene on the plasmid and to the restoration of a significant carbapenem-hydrolyzing phenotype.

## Introduction

*Atlantibacter* (formerly *Escherichia*) *hermannii* now belongs to the distinct genus *Atlantibacter* in Enterobacterales (Brenner et al., 1982; Hata et al., 2016). *A. hermannii* has rarely been implicated in clinical ocular infections (Poulou et al., 2008) or colonization of human wounds (Pien et al., 1985). *A. hermannii* harbors a chromosome-encoded class A β-lactamase (HER-1-like), conferring resistance to amoxicillin and ticarcillin, which is reversed by clavulanate, and a moderate susceptibility to piperacillin. But it remains susceptible to all cephalosporins and carbapenems (Fitoussi et al., 1995; Beauchef-Havard et al., 2003).

The VIM-1 enzyme is a metallo-β-lactamase (MBL) identified for the first time in 1997, in a carbapenem-resistant *Pseudomonas aeruginosa* isolate at the Verona University Hospital (Lauretti et al., 1999). This enzyme exhibits a very broad substrate specificity, including carbapenems, and was found to be encoded by a determinant carried on a mobile gene cassette inserted into an integron located on the chromosome or on plasmids (Di Pilato et al., 2014, 2015). Infection with VIM-1-producing *Klebsiella pneumoniae* has become endemic in some European countries, especially in intensive care units of tertiary care hospitals in Greece (Souli et al., 2008), and sporadic cases of infection due to multidrug-resistant Enterobacterales carrying *bla*_VIM–1_ have also been reported (Perilli et al., 2002; Souli et al., 2008).

PCR-based replicon typing allowed a rapid and accurate classification of plasmid families (Carattoli et al., 2005). The IncA/C plasmid family is one the largest resistance plasmid families along with IncFI, IncFII, IncN, IncH, IncX, IncL, and IncM (Carattoli, 2009). This plasmid family is strongly associated with resistance genes such as *bla*_CMY–2_ (Carattoli et al., 2012; Martin et al., 2012) and MBL encoding genes like *bla*_NDM–1_ or *bla*_VIM–1_ (Drieux et al., 2013; Esposito et al., 2017). This plasmid family was recently divided into two plasmid families IncA and IncC, formerly IncA/C1 and IncA/C2, respectively (Harmer and Hall, 2015). Compatibility of IncA and IncC has been recently demonstrated, reinforcing the fact that IncA and IncC plasmids belong to distinct groups (San Millan et al., 2015; Ambrose et al., 2018).

Several studies have shown that, despite the presence of the VIM-1 MBL in Enterobacterales, carbapenem MICs may remain under resistance thresholds (Psichogiou et al., 2008; Souli et al., 2008; Falcone et al., 2009, 2010). However, these isolates containing VIM-1 usually possess detectable carbapenemase activity using the Carba NP test (Dortet et al., 2012). The aim of this study was to decipher the discrepancy between the molecular detection of *bla*_VIM–1_ and carbapenem susceptibility confirmed by the absence of carbapenem-hydrolyzing activity in *A. hermannii*.

## Materials and Methods

### Bacterial Strains, Antimicrobial Susceptibility, and Carbapenemase Detection

*Enterobacter cloacae* complex WEB-1 and *A. hermannii* WEB-2 clinical isolates were collected from a rectal swab of a hospitalized patient in France, in 2015. Both isolates were first identified by MALDI-TOF mass spectrometry (MALDI Biotyper, Bruker, Wissembourg, France) and then by whole-genome sequencing (WGS) and comparison with genomes in GenBank data. Isogenic imipenem-resistant *A. hermannii* WEB-3 mutant was obtained by repeated cultures with increased concentrations of imipenem from 0.5 to 10 mg/L (0.5, 1, 2, 4, and 10 mg/L) of the *A. hermannii* WEB-2 clinical isolate. These repeated cultures correspond to one overnight culture in each imipenem-supplemented broth that was inoculated in the next twofold-dilution imipenem-supplemented broth. Except for cefiderocol, the MICs were determined by E-test (bioMérieux, France) and interpreted according to the CLSI breakpoints (Humphries et al., 2018). Cefiderocol MICs were performed according to EUCAST guidelines as updated in 2019 using broth microdilution plates (EUMDROXF Plate, Thermo Fisher). *Escherichia coli* TOP10 (Thermo Fisher Scientific, France) and *E. cloacae* CIP 7933 (Pasteur Institute Collection, Paris, France) were used as hosts for electroporation experiments and analysis of antimicrobial patterns. The updated Carba NP test (Nordmann et al., 2012; Dortet et al., 2014; Dortet et al., 2018) and MALDI-TOF mass spectrometry-based assays (MBT STAR^®^*-*Carba, Bruker) (Hrabák et al., 2012; Dortet et al., 2018) were performed as previously described to monitor carbapenem-hydrolyzing activity.

### Cloning of Naturally Occurring HER-9 Class A β-Lactamase of *A. hermannii*

The class A β-lactamase gene *bla*_HER–9_ was PCR amplified by PCR and sequenced with primers HER-9F and HER-9R, designed using the whole sequence of the WEB-2 isolate (Table 1). The obtained amplicon was cloned into the pCR-Blunt II-TOPO plasmid (Invitrogen). The recombinant pTOPO-HER-9 plasmid was electroporated into the *E. coli* TOP10, and the resulting recombinant *E. coli* TOP10 p(HER-9) was selected on ampicillin 100 mg/L containing trypticase soy agar (TSA) plates.

**TABLE 1:** Nucleotide sequences of primers used for amplification and sequence analysis.

Name	Sequence (5′–3′)	Size of the amplicon (bp)
rt-VIM-1F	ATTGTCCGTGATGGTGATGAG	132
rt-VIM-1R	ATGAAAGTGCGTGGAGACTGC	
rt-rpoBF	TGCCTGCAACCATCATTCTGC	106
rt-rpoBR	ATTTGCAGCTTGTTGTCACGG	
rt-repA-IncACF	AGCACGAAGACCTGTCCAAC	121
rt-repA-IncACR	TAGATCAGCACGGTTCTCC	
HER-9F	AATATCGGATAGAGTGGCGAGG	994
HER-9R	AATGCGCTTAAGCAGATTGGCG	

### Plasmid Extraction and Transformation

Plasmid DNA was extracted from both *E. hoffmannii* WEB-1 and *A. hermannii* WEB-2 isolates with the Kieser technique as previously described (Kieser, 1984) and electroporated in *E. coli* TOP10 and *E. cloacae* CIP 7933. Recombinant strains were selected on TSA supplemented with 100 mg/L of ticarcillin (Sigma, St-Quentin-Fallavier, France).

### Nucleic Acid Extractions, PCR, and qRT-PCR

Total DNA was extracted using the QIAGEN TipG500 genomic extraction kit (Qiagen, Courtaboeuf, France). The DNA concentration and purity were controlled by a Qubit^®^ 2.0 fluorometer using the dsDNA HS and/or BR assay kit (Life Technologies). Transcription of the carbapenemase gene was further studied by qRT-PCR using CFX96 (Bio-Rad) on RNA extracts from exponential phase (RNA minikit, Qiagen, Courtaboeuf, France) treated with DNAse. For each reaction, 20 ng of RNA was used. Transcription levels were standardized relative to the transcription level of the constitutive *rpoB* gene or to the *repA* gene located on the IncA plasmid (Table 1). The Rotor Gene SYBR Green OneStep RT-PCR kit (Qiagen) was used in qRT-PCR experiments, and all experiments were performed in triplicate. After the reverse transcription step for 10 min at 55°C, qPCR included an initial denaturation step of 3 min at 95°C, 40 cycles of denaturation (95°C/5 s) and annealing and extension (60°C/10 s) as previously described (Naas et al., 2012). The transcriptional levels were interpreted using 2^–ΔΔCt^ (Falcone et al., 2009; Girlich et al., 2017).

### Whole-Genome Sequencing and Genomic Analyses

Whole-genome sequencing was performed with two different techniques: short-read Illumina sequencing and long-read Oxford Nanopore technology. The DNA library was prepared either (i) by using the NEB Ultra II FS DNA library prep kit for Illumina (NEB, Evry, France) and run as 75- or 150-bp paired-end reads with V2 chemistry on a MiSeq sequencer or (ii) by using a R9.4 flow cell and the 1D native barcoding genomic DNA kit (SQK-LSK109) on a MinION sequencer (Oxford Nanopore Technologies, ONT). *De novo* assembly of the reads obtained with the libraries and comparison of the coverage of *bla*_VIM–1_, *repA* (IncA), *traI*, *parA*, *bla*_SHV–12_, and *intI1* genes from the plasmid and *rpoB*, *recA*, *dnaA*, *G6Pdh*, and *gyrB* genes from the chromosome were performed with CLC Genomics Workbench v12.1 (Qiagen, Les Ulis, France). Demultiplexing and assembly of the Nanopore reads were done with Guppy and Canu v1.8 workflows, respectively. The acquired antimicrobial resistance genes were identified using ResFinder server v2.1 (Center for Genomic Epidemiology (CGE^1^), and the genome was annotated using the RAST server. The plasmid content was analyzed by using PlasmidFinder (CGE) and manually searched by homology.

Representative members of all *Enterobacter* species have been used to classify *Enterobacter* spp. WEB-1, and a phylogenetic tree was created using the CSI Phylogeny 1.4 server^2^ (Kaas et al., 2014). Phylogenetic tree was represented using Figtree v1.4.3^3^.

### GenBank Accession Number

Genomes of *E. hormaechei* ssp. *hoffmannii* WEB-1 and *A. hermannii* WEB-2 and WEB-3 have been deposited in GenBank under GenBank accession number JAEVEU00000000, JAEVEV00000000, and JAEVEW00000000, respectively.

## Results and Discussion

### Bacterial Strains and Antimicrobial Susceptibility

Phylogenetic analysis revealed that *E. cloacae* complex WEB-1 actually corresponds to *E. hormaechei* ssp. *hoffmannii* WEB-1 (hereafter *E. hoffmannii* WEB-1) (Supplementary Figure 1). *A. hermannii* WEB-2 showed resistance to amoxicillin, ticarcillin, ceftazidime, and aztreonam, which was only slightly reversed by clavulanate; a moderate susceptibility to piperacillin and other cephalosporins; and susceptibility to carbapenems and cefiderocol (MICs at 0.12 mg/L). In contrast, *E. hoffmannii* WEB-1 was resistant to all β-lactams (Table 2). Regarding non-β-lactams, *A. hermannii* WEB-2 showed resistance to quinolones, sulfonamides, and trimethoprim–sulfamethoxazole and susceptibility to fosfomycin, gentamicin, chloramphenicol, and tigecycline (data not shown). The global susceptibility pattern suggested the production of an extended-spectrum-β-lactamase in addition to the chromosomal class A HER-type β-lactamase. WGS data confirmed the identification of the *bla*_SHV–12_ ESBL encoding gene. Analysis of the resistome of *E. hoffmannii* WEB-1 indicated the presence of three β-lactamase encoding genes: (i) *bla*_VIM–1_, (ii) *bla*_SHV–12_, and (iii) the cephalosporinase encoding gene *bla*_ACT–67_. Several aminoglycoside-modifying enzymes (AMEs) have been identified including *aadA1*, *aadA2-like*, *aph(3*″*)-Ib-like*, *aph(6*′*)-Id*, and *aac(6*′*)-Ib*. Acquired quinolone resistance *qnrS1* gene was also identified along with *dfrA14*, *sul1*, *sul2*, *catB2*, *cmlA1-like*, *mph(A)*, and *fosA*, encoding resistance to trimethoprim, sulfamethoxazole, chloramphenicol, macrolides, and fosfomycin, respectively. Noticeably, the *fosA* gene is naturally occurring in *Enterobacter* spp. The resistome of *A. hermannii* WEB-2 was similar to that of *E. hoffmannii* WEB-1, with the exception of the presence of *bla*_HER–like_ β-lactamase, the intrinsic class A β-lactamase of *A. hermannii*, and the absence of *cmlA1*-like and *bla*_ACT–67_. These close resistomes may indicate a probable plasmid location of the common genes and a conjugative transfer from one clinical isolate to the another (*E. hoffmannii* WEB-1 to *A. hermannii* WEB-2 or vice versa).

**TABLE 2:** MIC of β-lactams for *E. hoffmannii* WEB-1, *A. hermannii* WEB-2, isogenic mutant *A. hermannii* WEB-3, *E. coli* TOP10 wild type (WT), and recombinant *E. coli* isolates with plasmids from *E. hoffmannii* WEB-1 and from *A. hermannii* WEB-2 and for *E. cloacae* CIP7933 and recombinant *E. cloacae* isolates with plasmids from *E. hoffmannii* WEB-1 and from *A. hermannii* WEB-2.

	MIC of β-lactams (mg/L)									
	*E. hoffmannii*	*A. hermannii*	Isogenic mutant *A. hermannii*	*E. coli* TOP10				*E. cloacae* CIP 7933		
β-Lactam	WEB-1	WEB-2	WEB-3	WT	p(HER-9)	p(WEB-1)	p(WEB-2)	WT	p(WEB-1)	p(WEB-2)
Amoxicillin	>512	>512	>512	4	>512	>512	>512	128	>512	>512
Amoxicillin + CLA^a^	>512	64	64	1	16	> 512	>512	128	>512	>512
Ticarcillin	>512	>512	>512	1	>512	>512	>512	0.5	>512	>512
Ticarcillin + CLA	>512	>512	>512	1	16	>512	>512	0.5	>512	>512
Cefotaxime	128	128	128	0.25	0.12	64	64	0.25	128	64
Ceftazidime	>512	>512	>512	0.25	0.5	> 512	>512	1	128	128
Imipenem	4	0.5	1.5	0.25	0.25	2	2	1	4	4
Ertapenem	1	0.19	0.25	0.25	0.06	0.06	0.25	0.03	0.5	0.25
Meropenem	3	0.19	0.75	0.06	0.03	0.12	0.5	0.06	1	0.5

*^a^CLA, clavulanic acid at a fixed concentration of 4 mg/L.*

### Cloning and Sequencing of the HER-Type Class A β-Lactamase Gene

Cloning of the *bla*_HER–9_ gene in the pTOPO plasmid allowed us to obtain the recombinant *E. coli* TOP10 p(HER-9) strain expressing a narrow-spectrum resistance pattern limited to penicillins and susceptibility to β-lactamase/inhibitor combinations and to other β-lactams (Table 2). The deduced penicillinase HER-9 showed 79% amino acid identity with the closest relative, HER-6 from *A. hermannii* 2-89 (Beauchef-Havard et al., 2003). Contrary to the eight previously reported variants showing little inter-strain variability (≥98% identity) (Beauchef-Havard et al., 2003), this β-lactamase exhibited lower identity of 77.2–78.6%.

### Carbapenemase Activity

A significant carbapenemase activity could be detected for the *E. hoffmannii* WEB-1 using both the Carba NP test and the MALDI-TOF mass spectrometry-based assay, MBT STAR^®^*-*Carba assay. Intriguingly, on the *A. hermannii* WEB-2 isolate, the Carba NP test failed to detect any carbapenemase activity. Of note, the MBT STAR^®^*-*Carba assay was still able to detect a slight imipenem hydrolysis. Although not quantitative, this test already showed a higher sensitivity for the detection of carbapenemase with low hydrolytic activity (e.g., OXA-244) compared to biochemical tests (i.e., Carba NP test) (Hoyos-Mallecot et al., 2017).

### Plasmid Extraction, Transformation, and Expression

Plasmid DNAs from both *E. hoffmannii* WEB-1 and *A. hermannii* WEB-2 were extracted and transformed into either *E. coli* TOP10 or *E. cloacae* CIP7933 in order to compare the susceptibility profiles. Both recombinant strains expressed a phenotype compatible with the production of VIM-1 and SHV-12 β-lactamases and similar MICs to β-lactams (Table 2). In both strains, imipenem hydrolysis was successfully detected using the Carba NP test and MBT STAR^®^*-*Carba assay. This result demonstrated that the lack of detection of carbapenemase activity using the Carba NP test in *A. hermannii* WEB-2 was not due to the structure of the plasmid p(WEB-2) but rather to the genetic background of the parental strain.

### The *bla*_VIM–1_ Gene and the Genetic Context

In order to verify that differential expression was not due to different promoter sequences, upstream regions of *bla*_VIM–1_ were analyzed. In *E. hoffmannii* WEB-1 and *A. hermannii* WEB-2, the *bla*_VIM–1_ gene was enclosed in a common class 1 integron in first position. In both isolates, the *bla*_VIM–1_ gene was preceded by the P_c_H1 promoter [−35 (TGGACA) and −10 (TAAACT) regions spaced by 17 bp] and a P_2_ promoter, located 90 bp downstream of P_c_ [−35 (TTGTTA) and −10 (TACAGT) regions]. However, the promoter P_2_ might be inactive since −35 and −10 boxes were separated by only 14 bp. The third promoter P_int_ (TTGCTG-n = 17-TAGACT) is responsible for the expression of *intI1*. An inverse correlation has been demonstrated between the strength of promoter PC+P2 and excision activity of integrase (Jové et al., 2010). The association of PcH1 and inactive P2 gives rise to a weak promoter combination and thus may lead to a high excision activity of class 1 integrase. The same genetic structure was present at the promoter site in *E. hormaechei* WEB-1 and *A. hermannii* WEB-2 excluding any promoter-dependent differential expression of *bla*_VIM–1_ in these two isolates. Moreover, comparison of the same region between *A. hermannii* WEB-2 and its isogenic mutant *A. hermannii* WEB-3 showed no difference as well.

### Genomic and Transcriptomic Analysis of *E. hoffmannii* WEB-1, *A. hermannii* WEB-2, and *A. hermannii* WEB-3

An isogenic mutant of *A. hermannii* WEB-2, named *A. hermannii* WEB-3, was obtained after sequential passages of the parental strain in imipenem-containing broth. Although *A. hermannii* WEB-3 was able to grow on agar plates and in broth supplemented with 10 mg/L of imipenem; the carbapenem MICs were only slightly increased as compared to the parental strain and remained in the susceptible range (Table 2). Noticeably, the MIC of cefiderocol was increased in the mutant from 0.12 to 1 mg/L for *A. hermannii* WEB-2 and *A. hermannii* WEB-3, respectively. The addition of EDTA restored the susceptibility to cefiderocol at 0.25 mg/L (Supplementary Figure 2). Another crucial difference between *A. hermannii* WEB-2 and *A. hermannii* WEB-3 resides in the fact that *A. hermannii* WEB-3 mutant now possesses a detectable carbapenem-hydrolyzing activity using the Carba NP test.

WGS was performed simultaneously with DNA extracts from *E. hoffmannii* WEB-1, *A. hermannii* WEB-2, and the isogenic mutant *A. hermannii* WEB-3 using Illumina technology. Raw data are summarized in Supplementary Table 1.

Analysis of the genetic background (chromosome sequence) of the *A. hermannii* WEB-3 mutant strain vs. its parental *A. hermannii* WEB-2 isolates showed only one SNP in the *fliC* gene (T136 G) conferring an amino acid change in the flagellin (FliC) protein (Ser46Ala).

PlasmidFinder identified an IncA plasmid that is 98.32% identical to the reference IncA/C pRA-1 sequence (accession number FJ705807) in both *E. hoffmannii* WEB-1 and *A. hermannii* WEB-2 clinical isolates. Mapping of the reads of p(WEB-2) to p(WEB-1) used as the reference showed no difference between those IncA plasmids. In *E. hoffmannii* WEB-1, an additional plasmid belonging to the IncFIB subgroup was identified (data not shown). Analysis of the promoter sequence of *bla*_VIM–1_ on p(WEB-3) showed no mutation that could be involved in this differential expression of *bla*_VIM–1_ in the WEB-3 isogenic strain. Quantitative RT-PCR experiments performed on *E. hoffmannii* WEB-1, *A. hermannii* WEB-2, and *A. hermannii* WEB-3 isolates showed that the *bla*_VIM–1_ transcription level was similar in *E. hoffmannii* WEB-1 and *A. hermannii* WEB-2 when the *repA(IncA)* replicase gene was used as reference (22.1- vs. 25.4-fold of the expression level of *repA*). This result suggested a similar expression in isolates WEB-1 and WEB-2 when compared to *repA* expression. However, when *rpoB* is used as control, the expression of *bla*_VIM–1_ is 10-fold lower in WEB-2, indicating an overall lower expression of the carbapenemase in *A. hermannii* WEB-2. This result showed a weaker transcription relative to chromosomal genes in the parental *A. hermannii* WEB-2 strain (Table 3). Moreover, comparison of the transcription of *repA(IncA/C)* vs. that of *rpoB* showed that it was 10-fold higher in *E. hoffmannii* WEB-1 than in the *A. hermannii* WEB-2 isolate. On the other hand, the expression level of *bla*_VIM–1_ was 20-fold higher (406.7-fold of the expression level of *repA*) in *A. hermannii* WEB-3 (Table 4). Altogether, these qRT-PCR results showed that a weaker expression of VIM-1 in *A. hermannii* WEB-2 might be due to a lower replication of the IncA plasmid in *A. hermannii* (Table 4).

**TABLE 3:** Total read count and average coverage for chromosomal and plasmid-located genes in *E. hoffmannii* WEB-1, *A. hermannii* WEB-2, and isogenic mutant *A. hermannii* WEB-3.

	Total read count			Average coverage (x)		
Genes	*E. hoffmannii* WEB-1	*A. hermannii* WEB-2	*A. hermannii* WEB-3	*E. hoffmannii* WEB-1	*A. hermannii* WEB-2	*A. hermannii* WEB-3
**Plasmid-located genes**						
*repA*	2,724	1,890	232	185.55	129.02	31.68
*parA*	1,873	1,262	194	179.23	120.55	37.08
*traI*	5,203	3,670	895	131.87	93.15	45.38
*bla*_VIM–1_	1,441	1,114	4,761	135.09	104.54	891.63
*bla*_SHV–12_	1,252	976	6,684	100.09	77.95	1,069.11
*intI1*	6,350	2,680	9,099	470.59	198.73	1,348.32
**Chromosomal genes**						
*dnaA*	1,148	1,256	1,098	61.93	72.05	125.66
*rpoB*	4,810	4,868	1,496	85.62	90.95	55.79
*recA*	883	1,108	651	62.83	78.48	91.69
*Glucose 6-phosphate dehydrogenase*	1,199	1,304	952	37.44	66.37	96.92
*gyrB*	1,179	2,429	2,062	36.83	75.43	128.24

**Coverage**	***E. hoffmannii* WEB-1**		***A. hermannii* WEB-2**		***A. hermannii* WEB-3**	

Mean chromosome	56.93	± 20.41	76.66	± 9.17	99.66	± 29.53
Mean plasmid	165.55	± 29.33	114.24	± 18.75	38.05	± 6.90
Integrase	470.59		93.15		1,348.32	
VIM-1	135.09		104.54		891.63	
Ratio plasmid/chromosome	2.91		1.49		0.38	
Ratio VIM-1/plasmid	0.82		0.92		23.44	
						

**TABLE 4:** Fold change RNA transcription of *bla*_VIM–1_ relative to that of *repA(IncA)* and *rpoB* in *E. hormaechei* WEB-1, *A. hermannii* WEB-2, and isogenic mutant *A. hermannii* WEB-3.

Strain	Fold change expression (2−^ΔΔ*Ct*^) (± SD)^a^		
	*bla*_VIM–1_ vs. *repA(IncA)*	*bla*_VIM–1_ vs. *rpoB*	*repA(IncA)* vs. *rpoB*
*E. hormaechei* WEB-1	22.1 (± 1.5)	6.0 (± 0.3)	0.27 (± 0.03)
*A. hermannii* WEB-2	25.4 (± 1.8)	0.6 (± 0.1)	0.03 (± 0.003)
Isogenic mutant *A. hermannii* WEB-3	406.7 (± 35)	6.3 (± 1.3)	0.02 (± 0.002)

*^a^SD, standard deviation.*

### Genomic Analysis of *E. hoffmannii* WEB-1, *A. hermannii* WEB-2, and Its Mutant: Role of Gene Amplification and Plasmid Copy Number

**FIGURE 1:**
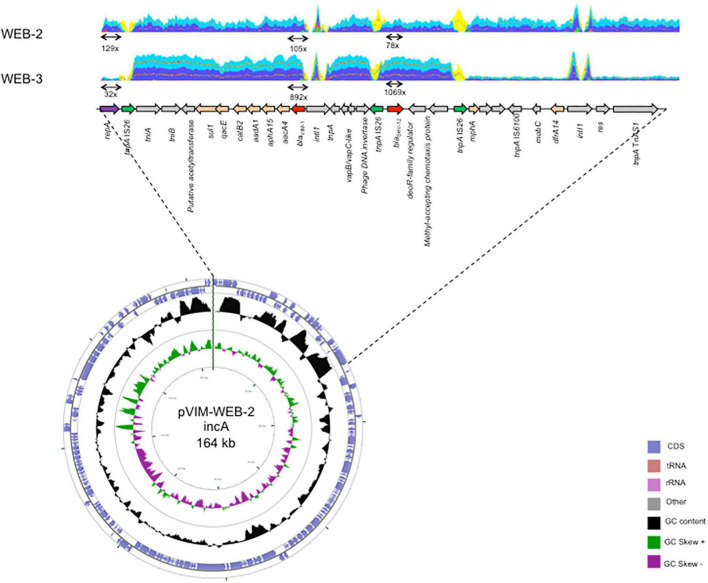
Schematic representation of the p(WEB-2) plasmid and the structure carrying the *bla*_VIM–1_ carbapenemase gene in *A. hermannii* WEB-2 and WEB-3. Open reading frames are indicated by arrows. Gene coverage was indicated at scale with the genetic organization. Gene coverage is indicated on selected features and determined using Illumina read sequencing.

To confirm qRT-PCR results, comparison of the coverage of the plasmid-located genes (*bla*_VIM–1_, *repA(IncA)*, *traI (IncA)*, and *bla*_SHV–12_ *and intI1*) vs. that of chromosomal genes (*rpoB*, *recA*, *dnaA*, *g6-pdh*, and *gyrB*) highlighted that plasmid genes were covered 2.91-fold more than those located on the chromosome in *E. hoffmannii* WEB-1, whereas this coverage was only 1.49-fold in *A. hermannii* WEB-2 (Table 3). It suggests that *E. hoffmannii* WEB-1 harbors twofold more copies of the *bla*_VIM–1_-carrying IncA-type plasmid compared to *A. hermannii* WEB-2. Of note, the coverage ratio *bla*_VIM–1_/plasmid is nearly identical in *E. hoffmannii* WEB-1 and *A. hermannii* WEB-2 (0.82 vs. 0.92), suggesting that only one copy of *bla*_VIM–1_ was present on p(WEB-1) and p(WEB-2) plasmids. Quantitative RT-PCR experiments showed that the transcription level of *bla*_VIM–1_ was similar in *E. hoffmannii* WEB-1 and *A. hermannii* WEB-2 when the *repA(IncA)* replicase gene was used as reference (22.1 vs. 25.4-fold the expression level of *repA*) (Table 4). It confirms an equal copy number of *bla*_VIM–1_ on both plasmids p(WEB-1) and p(WEB-2), previously deciphered as one copy per plasmid by coverage analysis (Table 3). On the contrary, the expression level of *bla*_VIM–1_ was ∼20-fold higher (406.7-fold the expression level of *repA*) in *A. hermannii* WEB-3 compared to *E. hoffmannii* WEB-1 and *A. hermannii* WEB-2 (Table 4 and Figure 1). It might be due to (i) ∼20 duplications of *bla*_VIM–1_ containing elements on the p(WEB-2) plasmid, (ii) to duplication of such element on the (WEB-3) plasmid as well as on the chromosome, or (iii) to the higher copy number of the *bla*_VIM–1_-carrying plasmid in the *A. hermannii* WEB-3 strain. As shown in Table 4, no increase of plasmid copy number was evidenced in the *A. hermannii* WEB-3 strain but rather a decrease of copy number. In addition, a striking increase of *bla*_VIM–1_/plasmid coverage was observed from 0.92 to 23.44 X (Table 3). Accordingly, this result is in favor of ∼20 duplications of *bla*_VIM–1_-containing elements on the p(WEB-3) plasmid including the whole integron and surrounding region. In addition, the long-read WGS analysis confirmed that *bla*_VIM–1_ was absent from the chromosomes of *A. hermannii* WEB-2 and *A. hermannii* WEB-3.

Since the mean size of MinION reads was 6 kb (Supplementary Table 1), these multiple duplications (∼20 duplications) did not allow us to reconstruct the entire p(WEB-3) plasmid despite long-read WGS having been performed. Thus, to decipher if only *bla*_VIM–1_ or the complete integron was duplicated on the p(WEB-3) plasmid, Illumina reads of *A. hermannii* WEB-3 were mapped against p(WEB-2), which was easily reconstructed from the long-read WGS data of *A. hermannii* WEB-2 (Figure 1). Coverage analysis indicated that the *bla*_VIM–1_ gene cassette was not the sole duplicated gene. The duplicated region was a 17-kbp fragment bracketed by two copies of IS*26* (Figure 1). Of note, due to the size of MinION reads, only two copies of this entire region were evidenced on a single read. To verify that the p(WEB-3) plasmid corresponded to an increased size of 391 kbp (corresponding to 23 kbp × 17 kbp) compared to the p(WEB-2) plasmid, an extraction was performed followed by gel electrophoresis. Unfortunately, despite repeated attempts, no size estimation was possible. Indeed, the p(WEB-3) plasmid extracted by the Kieser method did not migrate properly in the 0.7% agar gel likely due to its huge size.

## Conclusion

Here, we described the identification of the *bla*_VIM–1_ gene in *A. hermannii* that weakly expressed its carbapenemase. In the same patient, an *E. hoffmannii* was previously recovered and correctly identified as a VIM-1 producer. Analysis of the plasmid content of these two isolates revealed a similar *bla*_VIM–1_-carrying plasmid belonging to the IncA-incompatibility group. Both plasmids were transferred in *E. coli* and showed similar resistance profiles when introduced in the same genetic background. The analysis of genetic coverage coupled with qRT-PCR experiments led us to decipher that the weaker expression of *bla*_VIM–1_ in *A. hermannii* WEB-2 as compared to *E. hoffmannii* WEB-1 was the consequence of a lower *bla*_VIM–1_-carrying plasmid copy number in *A. hermannii* WEB-2. Upon imipenem exposure, we selected an isogenic mutant with higher carbapenem MICs and a significant carbapenemase activity. In this mutant, the plasmid copy number was lower than that in the parental strain, but the region encompassing the carbapenemase gene, bracketed by copies of IS*26*, was duplicated several times. It highlights the ability of *A. hermannii* to respond to antibiotic selective pressure by duplicating antimicrobial resistance gene copy numbers. Of note, we identified that these multiple duplications occurred in a region bracketed by IS*26* inside the parental plasmid. As previously described, it highlights the role of IS*26* in gene amplification through hot spot duplication (Hubbard et al., 2020; Rodríguez-Villodres et al., 2020).

Finally, despite the fact that *A. hermannii* and *E. hoffmannii* belong to the family of Enterobacteriaceae (Enterobacterales order), the expression and replication of plasmids may vary from one genus to another, leading to altered phenotypes that result in the misidentification of carbapenemase-encoding genes. In addition, we demonstrated that under specific antibiotic selective pressure, clinical isolates possessing carbapenemase-encoding genes without phenotypic expression could easily convert to true carbapenemase producers (increase of MICs and carbapenemase activity) through gene duplication as observed for ceftazidime/avibactam resistance in *K. pneumoniae* (Coppi et al., 2020).

## Data Availability Statement

The datasets presented in this study can be found in online repositories. The names of the repository/repositories and accession number(s) can be found below: https://www.ncbi.nlm.nih.gov/genbank/, JAEVEU00000000; https://www.ncbi.nlm.nih.gov/genbank/, JAEVEV00000000; and https://www.ncbi.nlm.nih.gov/genbank/, JAEVEW00000000.

## Author Contributions

DG and RB: study design, analysis, and proofreading of manuscript. AP: data analysis. TN: proofreading of manuscript. LD: study design and proofreading of manuscript. All authors contributed to the article and approved the submitted version.

## Conflict of Interest

The authors declare that the research was conducted in the absence of any commercial or financial relationships that could be construed as a potential conflict of interest.

## Publisher’s Note

All claims expressed in this article are solely those of the authors and do not necessarily represent those of their affiliated organizations, or those of the publisher, the editors and the reviewers. Any product that may be evaluated in this article, or claim that may be made by its manufacturer, is not guaranteed or endorsed by the publisher.

## Footnotes

1
https://cge.cbs.dtu.dk/services/

2
https://cge.cbs.dtu.dk/services/CSIPhylogeny/

3
http://tree.bio.ed.ac.uk

## Supplementary Material

Supplementary Figure 1 Phylogenetic tree of *Enterobacter* species. Accession number of representative isolates of each species is indicated within brackets. *Enterobacter* WEB-1 is indicated in bold. This phylogenetic tree was obtained using CSI Phylogeny.

Supplementary Figure 2 Impact of *bla*_VIM–1_ gene amplification on susceptibility to cefiderocol, mecillinam, moxalactam, and cefoxitin of *A. hermannii* WEB-2 and WEB-3. Seven microliters of EDTA at 0.5 M was used for inhibition.
