# Supplementary figures and images for: Undetectable Production of the VIM-1 Carbapenemase in an Atlantibacter hermannii Clinical Isolate
Source: Front Microbiol. 2021 Dec 20;12:741972. doi: 10.3389/fmicb.2021.741972 (PMC8721206; doi:10.3389/fmicb.2021.741972)

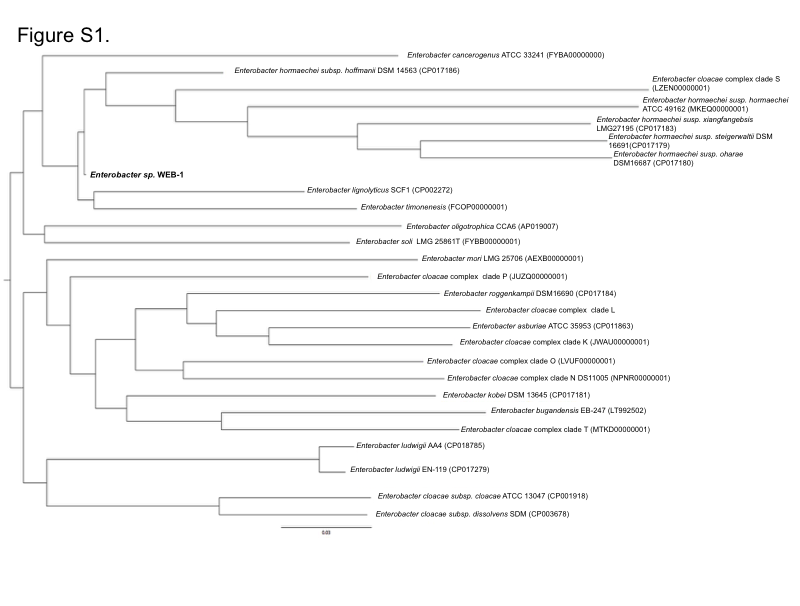

Supplement: Supplementary Figure 1 — Phylogenetic tree of Enterobacter species. Accession number of representative isolates of each species is indicated within brackets. Enterobacter WEB-1 is indicated in bold. This phylogenetic tree was obtained using CSI Phylogeny. [file Image_1.TIFF]

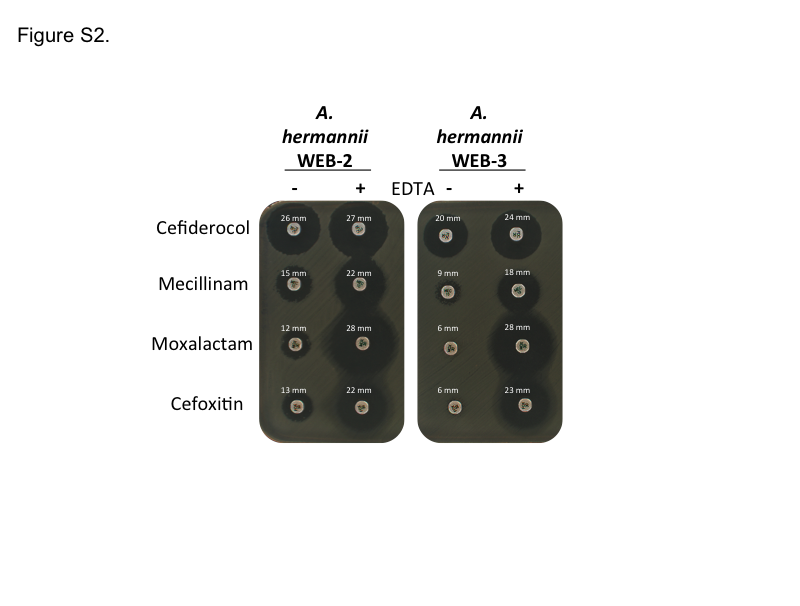

Supplement: Supplementary Figure 2 — Impact of blaVIM–1 gene amplification on susceptibility to cefiderocol, mecillinam, moxalactam, and cefoxitin of A. hermannii WEB-2 and WEB-3. Seven microliters of EDTA at 0.5 M was used for inhibition. [file Image_2.TIFF]
