# Supplementary material for: Undetectable Production of the VIM-1 Carbapenemase in an Atlantibacter hermannii Clinical Isolate
Source: Front Microbiol. 2021 Dec 20;12:741972. doi: 10.3389/fmicb.2021.741972 (PMC8721206; doi:10.3389/fmicb.2021.741972)
Supplement: Supplementary file 3 [file Table_1.DOCX]

**SUPPLEMENTARY DATA**

Table S1. Sequencing results

| Isolates | Sequencing technology | Read size | Number of reads | Number of total nucleotides | Number of contigs | N50 (bp) |
| --- | --- | --- | --- | --- | --- | --- |
| *E. hoffmannii* WEB-1 | Illumina | 75 bp | 5 620 310 | 422 551 051 | 125 | 94,200 |
| *A. hermannii* WEB-2 | Illumina | 150 bp | 1 868 880 | 251 803 344 | 51 | 186,696 |
| *A. hermannii* WEB-3 | Illumina | 150 bp | 2 966 268 | 390 258 664 | 130 | 83,151 |
| *A. hermannii* WEB-3 | MinIon | 6,043 bp^a^ | 17,010 | 102 803 941 | 1 |  |

^a^Mean size of MinIon reads
